# Understanding Loneliness in Brain Injury: Linking the Reaffiliation Motive Model of Loneliness With a Model of Executive Impairment

**DOI:** 10.3389/fnint.2022.883746

**Published:** 2022-07-14

**Authors:** Christopher Byrne, Christian E. Salas, Rudi Coetzer, Richard Ramsey

**Affiliations:** ^1^School of Human Behavioural Sciences, Bangor University, Bangor, United Kingdom; ^2^North Wales Brain Injury Service, Colwyn Bay Hospital, Colwyn Bay, United Kingdom; ^3^Centre for Research in Human Neuroscience and Neuropsychology, Faculty of Psychology, Diego Portales University, Santiago, Chile; ^4^Clinical Neuropsychology Unit, Faculty of Psychology, Diego Portales University, Santiago, Chile; ^5^College of Human and Health Sciences, Swansea University, Swansea, United Kingdom; ^6^The Disabilities Trust, Wakefield, United Kingdom; ^7^Department of Psychology, Macquarie University, Sydney, NSW, Australia

**Keywords:** acquired brain injury (ABI), executive impairments, loneliness, neuropsychological rehabilitation, social isolation

## Loneliness and Brain Injury

In the early history of human evolution, the need to belong to others may have been protective as those who were on the periphery of social groups may have been less likely to survive. Group participation likely ensured more access to food, shelter, protection from external threat, and increased opportunity to mate (Hienrich and Gullone, 2006). It has been proposed that, through processes of natural selection, humans have evolved the need for social connectedness at both a biological and psychological level (Masi et al., 2011). Thus, the subjective feeling of loneliness has been proposed as an adaptive form of ‘social pain' which motivates humans to seek social connection (Eisenberger, 2012).

Although *transient* loneliness may be considered adaptive, studies have demonstrated that persistent loneliness can become maladaptive, resulting in various negative outcomes across both psychological and physical domains (Cacioppo and Patrick, 2008; Holt-Lunstad et al., 2015; Hakulinen et al., 2018). Several reports have established positive correlations between loneliness and increased morbidity. For example, the deleterious effects of loneliness have been shown to be comparable with other well-known clinical risk indicators such as smoking, and even greater to other factors such as obesity and high cholesterol (Pantell et al., 2013). Large meta-analytical studies have demonstrated that those who live alone, are socially isolated and subjectively feel lonely demonstrate an average 26–32% increased likelihood of mortality (Holt-Lunstad et al., 2015). The mechanisms that underpin the deleterious effects of loneliness, such as increased morbidity, are not yet fully established, but are likely to span a range of physiological systems (Friedler et al., 2015). A detailed review on recent neurophysiological models of loneliness is outlined in Quadt et al. (2020), and summarized by Gronewold and Engels (2022). Additionally, a large cohort study using the UK biobank data (Hakulinen et al., 2018), highlights the association between social isolation and mortality, which may be mediated by a reduction in access to support during illness.

The terms loneliness and social isolation have been often used interchangeably. However, they should be considered as two distinct concepts. Social isolation refers to a decreased quantity of social relations with other people (Zavaleta et al., 2014). Studies often conceptualize the quantity of social contact as the *structural characteristics* of a social network (size, composition, frequency and length of contact). In contrast, the quality of social contact has been defined as the individual's subjective assessment of how satisfied they are with their social relationships. Importantly, the qualitative interpretation that your social needs are not being met is the hallmark of loneliness. Moreover, several studies have shown that quantitative and qualitative components of social relationships are dissociable (Salas et al., 2021; Byrne et al., 2022). For example, a person may have a small social network but experience it as supportive, or have a large social network and feel lonely.

Despite evidence regarding the presence and heterogeneity of loneliness across the life span (Qualter et al., 2015), the current literature is dominated by research examining loneliness in older normotypical/non-neurological populations, or those with progressive neurological conditions such as dementia. It has been noted nevertheless that individuals and groups with different forms of disability may be particularly vulnerable to social isolation (Durcan and Bell, 2015). People with different forms of neurological illnesses are especially vulnerable to social isolation due to participation limitations imposed by motor, cognitive and socio-emotional impairments. Furthermore, loneliness and social isolation have also been shown to mediate the trajectory of recovery, stagnation, or indeed deterioration, of neurological conditions (Glass and Maddox, 1992).

There is a growing literature reporting higher levels of loneliness in adults with acquired brain injury. Recent research (Byrne et al., 2022) demonstrated that 30–44% of those with a history of stroke report experiencing loneliness. Furthermore, those with a history of stroke were found to be 70% more likely to report loneliness when compared to the healthy individuals. To put this into perspective, amongst those that live with brain injury, loneliness has similar, if not greater, prevalence rate than other psychological complaints, such as depression (31%) and anxiety (20%) (Schöttke and Giabbiconi, 2015). Importantly, it has been reported that loneliness, and not the size of the network, or level of perceived social support, is the best predictor of quality of life, emotional wellbeing and depression in people with acquired brain injury (ABI) who live in the community (Salas et al., 2021).

Despite this emerging evidence, there is a lack of information regarding why people with ABI may feel *persistently* lonely, exploring the potential contribution of cognitive/behavioral deficits and interpersonal factors. Therefore, the goal of this article is to contribute to this discussion by linking two lines of research: loneliness and executive functioning. To do so, we introduce a model of loneliness (The Reaffiliation Motive (RAM) model; Qualter et al., 2015) as a useful theoretical framework that can be used to map how cognitive impairments may contribute to *persistent* loneliness after brain injury. In the following, we present the RAM model, its components and theoretical background. We then introduce the model of executive functions proposed by Stuss (2011), which describes different profiles of executive impairment: energization, executive cognition, emotion/behavioral regulation and metacognition. Each of these profiles is discussed in relation to the RAM model, underscoring how diverse profiles of neurocognitive impairment could compromise different stages of the reaffiliation process.

## In Search of a Model to Understand Loneliness After Brain Injury

There is currently a lack of models that aim to understand loneliness after brain injury. However, there are models developed to explain the experience of loneliness in normotypical individuals, which may be generalized to explore this problem in brain injury survivors. The Evolutionary Theory of Loneliness (ETL), proposed by Cacioppo and colleagues (Cacioppo and Cacioppo, 2018) suggests that the experience of loneliness, like physical pain, is ultimately an evolutionary protective mechanism - a type of social pain. Like pain, loneliness is an aversive experience, but with the primary purpose to motivate individuals to reintegrate and seek safety in the form of social connection. However, this model does not account for all individuals. For example, the Evolutionary Theory of Loneliness does not account for those who actively avoid social connection because of their anxiety or social phobia, or persons with personality structures driving them to avoid social contact.

**Figure 1:**
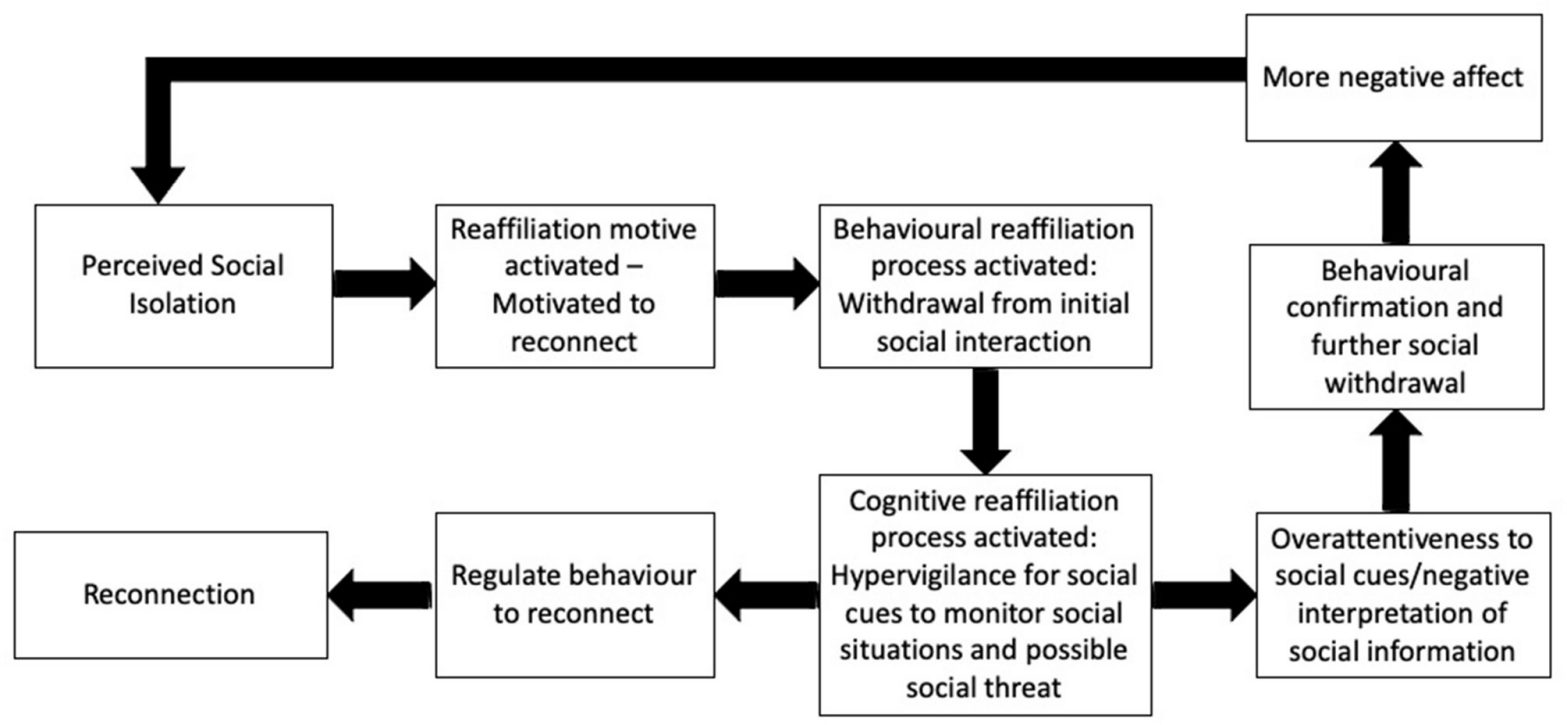
The reaffiliation motive (RAM) model of loneliness (Qualter et al., 2015).

The RAM model (Qualter et al., 2015) expands on the motivational aspect of Cacioppo's ETL. Like the ETL, the RAM model suggests that the experience of loneliness is an adaptive signal that *motivates* individuals to reconnect with other people. The RAM model describes specific components of the re-affiliation process, placing the *perception* of social isolation at the start (Figure 1). The perception of social isolation activates the *motivation to reconnect* (reaffiliation motive), which in turn results in a paradoxical behavioral response - *withdrawal*. The paradoxical withdrawal response refers to an increase in the motivation to connect with others but at the same time an increase in the implicit hypervigilance for social cues to monitor social interactions and social threats. This response allows individuals to appraise their own behavior and the social environment to analyze it for threats; thus, modifying cognitive-behavioral responses that may lead to either reaffiliation, ending the transient feeling of loneliness, or persistent loneliness. The direction of outcome is mediated by the cognitive-behavioral processes that take place. For example, prolonged loneliness is associated with maladaptive cognitive bias such as attentional bias, memory bias, attribution bias and external locus of control. These cognitive biases can compromise reaffiliation resulting in further behavioral withdrawal and subsequent negative affect.

The RAM model is a promising theoretical tool since it suggests that the *process* by which people reconnect to others when feeling lonely is complex and requires cognitive, behavioral and interpersonal resources. Consequently, it can be expected that individuals who present with cognitive, behavioral or interpersonal impairments after brain injury, will experience difficulties navigating through this process in order to reconnect. Acquired brain injury not only can generate loneliness, but more importantly, it can also compromise people's capacity to overcome loneliness by actively reconnecting with others, thus generating persistent loneliness. Because brain injured survivors can experience a wide and varied range of cognitive, behavioral and socio-emotional deficits, there are many ways in which reaffiliation can be compromised. In this article we use the case of executive impairment profiles as an example to show how specific neuropsychological deficits can compromise different components or tasks in the RAM model.

## Reaffiliation Failure in People With Executive Impairment

Executive Dysfunction is a common problem after several forms of ABI (e.g., stroke, traumatic brain injury), often -but not exclusively- related to prefrontal lobe damage. It has been defined as an impairment in a wide set of skills required for effective problem solving, planning and organization, self-monitoring, initiation, error correction and behavioral regulation (Evans, 2005). There is robust evidence reporting that executive impairment can cause devastating social handicap (McMillan and Wood, 2017). However, we know little about the potential relationship between executive impairment and loneliness after ABI. To our knowledge, there is only one study that has directly explored this link, showing a modest to strong correlation between executive dysfunction, as measured by the Frontal Systems Behavior Scale, and reported loneliness (Cristofori et al., 2019).

**Figure 2:**
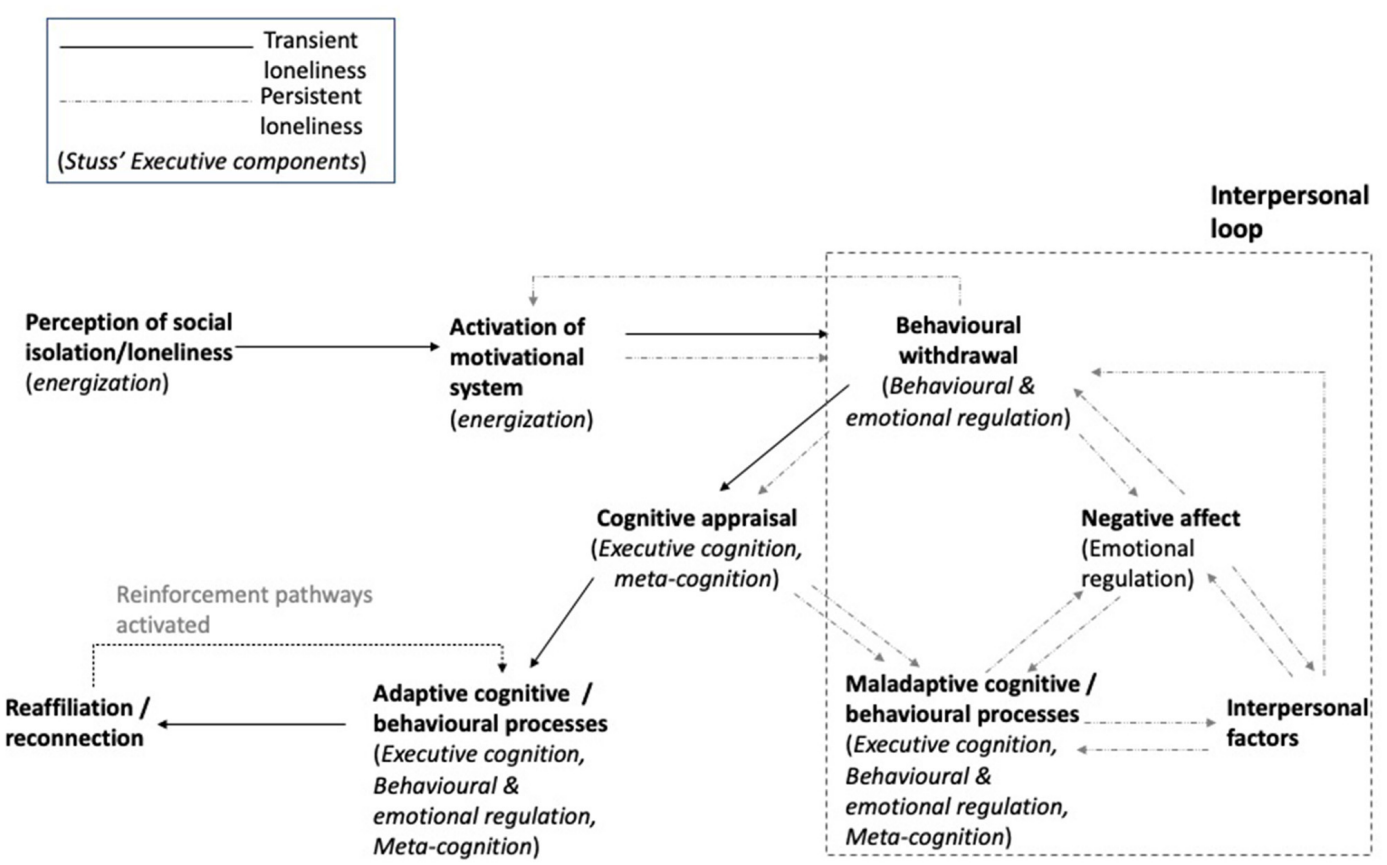
Executive components associated with the reaffiliation motive (RAM) model of loneliness. Components of the RAM model (Qualter et al., 2015) are outlined in bold font. In parentheses, we outline the executive components from Stuss's model (2011) that are likely to play a role in each aspect of the RAM model. The model begins with *energisation* as the foundational component, required to both perceive social cues and to activate the motivational system for the behavioral withdrawal process to begin. As described by Qualter et al. (2015), behavioral withdrawal allows individuals to appraise the social landscape. This requires *behavioral and emotional regulation* abilities. Once withdrawn, cognitive appraisal of the social situation occurs, requiring *executive cognitive* and *meta-cognitive* processes. It is at this stage where adaptive or maladaptive responses (cognitive and behavioral) begin to dictate the direction of travel along the model resulting in loneliness becoming transient, or persistent. Adaptive cognitive and behavioral processes, requiring *executive systems*, facilitate reaffiliation, which subsequently reinforces future adaptive cognitive and behavioral processes. In contrast, maladaptive cognitive and behavioral processes, through executive impairment, may result in persistent loneliness loop reinforced by both intrapersonal and interpersonal factors.

The *Model of Frontal Lobe Functioning* (Stuss, 2011) has been widely used in Neuropsychological Rehabilitation (Winson et al., 2017). It suggests that four executive components can be differentially compromised after brain injury: Energization, Executive Cognition, Emotional and Behavioral Self-Regulation and Metacognition. It can be argued that individuals with dysexecutive impairment can be clustered in four groups or profiles, each of them with a *predominant* deficit in one or more of these components. In this section we will describe the main clinical presentation of each profile and formulate hypotheses regarding how the RAM model and its components might be differentially compromised (Figure 2).

### Energisation

Energization has been defined as the capacity to internally initiate, and sustain, a voluntary or non-reflexive response. Energisation problems are common after damage to superior medial prefrontal structures (BA 24,9 and 6, Stuss, 2011) and have been related to amotivational syndromes such as apathy and abulia (Moretti and Signori, 2016; Henri-Bhargava et al., 2018). Individuals with energization problems often report they lack energy and struggle starting activities or sustaining a behavior in time, while relatives often describe a drastic lack of motivation and apparent laziness (Winson et al., 2017). In many cases, individuals with energization problems are mistakenly diagnosed as presenting a major depressive disorder due to loss of interest and energy. Patients with energization problems initiate speech or action less often than normal and can be slow in “getting a behaviour started”. Their thinking process can also be compromised, becoming less fluent and generative, often reporting the “blank mind” phenomenon. Consequently, these patients may take longer in accomplishing tasks, although their performance may present few mistakes.

Energization problems can compromise several reaffiliation components. Perhaps the most important one is the *perception of social isolation*, or social pain, which can be altered due to impairment in emotional reactivity. Emotional reactivity has been defined as an emotional process related to the activation of emotional responses, its intensity and duration (Becerra and Campitelli, 2013). Individuals with dorsomedial prefrontal cortex damage, particularly those with lesions to the Anterior Cingulate Cortex, have been classically described as presenting a dampened emotional reactivity (Siegel et al., 2014), as well as other related disorders, such as alexithymia (Schäfer et al., 2007), altered interoceptive awareness (Critchley, 2004) and altered pain detection (Xiao and Zhang, 2018). Consequently, and due to altered emotional reactivity, individuals with energization problems might appear impaired at triggering feelings of loneliness during the *perception of social isolation* phase. Due to apathy and altered behavior initiation, people with energization problems might also fail activating the *motivation to reconnect*, thus compromising the whole reaffiliation process. As noted before, energization problems can also alter cognition, by decreasing people's ability to generate mental contents. This impairment may be particularly relevant during the *cognitive reaffiliation phase*, where an appraisal of the social environment is needed in order to analyze information and determine a plan of action to reaffiliate successfully.

### Executive Cognition

Executive Cognition has been described as a process that involves both the capacity to develop and implement a plan when facing a novel task (task setting) and the ability to check that one remains on task over time adjusting behavior if needed (monitoring) (Henri-Bhargava et al., 2018). These abilities have been associated with damage to the left and right dorsolateral prefrontal cortex, respectively (BA 44, 45, 46, 9, 9/46, and 47/12, Stuss and Alexander, 2007). Individuals with task setting impairments often struggle to switch from one task to another, showing signs of inflexibility and perseveration, while brain injured survivors with monitoring problems often struggle recognizing mistakes. When executive cognition is altered, survivors may report difficulties coming up with new ideas, thinking outside the box, staying on track or getting things done. Relatives, on the other hand, often comment that their loved ones have become repetitive, concrete or stubborn (Winson et al., 2017).

Individuals with problems in executive cognition might struggle moving along the different phases of the reaffiliation process. They might experience significant difficulties during the *cognitive reaffiliation* phase, where the formulation of a social assessment is required. Due to a tendency to consider only what is apparent and can be seen, people with executive cognition impairments may struggle reading subtle and complex social interactions and mental states. People with executive cognition impairments can also struggle “seeing the forest and not just the trees”, thus, becoming stuck in one or two aspects when reading a social situation (Winegardner, 2017).

There is also evidence showing that individuals that tend to perseverate can become fixated on negative elements of a social situation, easily falling into a negative emotional and interpersonal loop and struggling to downregulate negative feelings on their own (Salas C. E. et al., 2013; Salas et al., 2014). As a consequence, many individuals with executive cognition problems can be over-attentive to negative social cues and negatively interpret social situations. In other words, they struggle with reaffiliating because social interaction is experienced as a threat, then emotional avoidance, minimization and distancing are employed as coping strategies (Krpan et al., 2007). The RAM model suggests that the information obtained during the cognitive reaffiliation phase is used to guide behavior during the *reconnection* phase. It is well-known that individuals with executive cognition problems may become disorganized or inflexible in their behaviors and ideas when dealing with unstructured and emotionally relevant situations (Sandson and Albert, 1984). They can also struggle monitoring their behavior (what works and what doesn't) and flexibly adapting it to environmental changes in order to successfully reconnect.

### Emotional and Behavioral Regulation

Emotional and behavioral self-regulation is a capacity related to the integration of motivational, reward/risk, emotional and social aspects of behaviors (Stuss, 2011). Damage to the ventromedial prefrontal cortex (BA 32, 25, 24, 14, 13, 12, 11) often compromises this ability (Henri-Bhargava et al., 2018) and has been commonly referred to as orbitofrontal syndrome (Chow, 2000). Individuals impaired in the regulation of behavior and emotion can struggle controlling thoughts, emotions and actions, and expressing them according to social conventions. They can experience marked emotion dysregulation, with emotions rising up quickly, with increased intensity and difficulty in controlling them. They are often described by relatives as impulsive, egocentric, doing things without thinking or behaving almost “childishly” or “child-like” (Winson et al., 2017).

Impairments in behavioral and emotional self-regulation would very likely compromise several components of the RAM. First, the *behavioral reaffiliation* component indicates that individuals initially withdraw from the immediate social environment once loneliness is perceived, in order to assess the level of social threat. Those with behavioral and emotion regulation difficulties may struggle inhibiting their behavior in order to withdraw, as a means to assess changes in a social situation. In other words, they may struggle stopping behavior in order to think about what is going on, thus tending to “act or speak without thinking”. For example, a survivor may become over familiar with people he/she just met, in order to manage feelings of isolation or exclusion during a social gathering.

Second, emotional dysregulation can compromise *cognitive reaffiliation* by strengthening perceptual, attentional and negative memory biases. Here, inhibitory failures are relevant to consider, since the inhibition of self-perspective is necessary to comprehend other people's emotions, desires and experiences, particularly those that differ from ours (Samson et al., 2005). As a consequence, survivors can attribute to others intentions based on their own negative mental states, thus actively contributing to negative social interactions. This can be further compounded by difficulties controlling emotional arousal. Those with such difficulties can experience emotions quickly and intensely. This can lead to situations where an individual's emotional response may be both immediate and over amplified, resulting in interpersonal conflict.

### Metacognition

Finally, metacognition has been defined as an integrative function that coordinates the other three executive components (Stuss, 2011). Metacognition can be compromised after damage to the frontal poles (BA 10s and 10i) and is often observed as a difficulty in observing one's own mental processes (self-awareness) and understanding other people's mental states, particularly when they differ (theory of mind) (Fleming et al., 2014). Individuals with damage in this PFC area can experience difficulties in understanding humor, reading their own and other people's emotions, taking someone else's point of view and being aware of their own abilities and deficits (Stuss et al., 2001). Relatives often describe a change in personality of those with metacognitive problems and, more importantly, a lack of recognition of those changes (Winson et al., 2017).

Higher-level metacognitive processes are likely required throughout the reaffiliation process. Therefore, when impaired, metacognitive difficulties can pose a barrier at several points of the model. The *perception* of loneliness may be compromised due to an inability to interpret and reflect on the social situation. Here, concrete patients are an illustrative example. Concreteness has been defined as a form of metacognitive impairment, characterized by a difficulty in detaching from immediate experience in order to observe and reflect upon emotions and mental states related to the self and others (Salas C. et al., 2013; Salas and Coetzer, 2015). It has been noted that concrete patients can experience emotional distress but struggle understanding the reasons behind their phenomenological experience (Coetzer, 2003). In other words, they can feel upset or low, but it is difficult for them to grasp that those emotions are related to feeling lonely. In the most extreme form, an individual may lack total awareness of their social situation, which is pivotal to start the RAM process.

Metacognitive processes are also likely to be important for the *cognitive reaffiliation* component of the RAM model. Individuals with metacognitive difficulties may be unable to reflect on their behavior and thoughts, thus perpetuating maladaptive cognitive and behavioral responses that led to social isolation. For example, individuals with egocentricity may be unable to see the perspective of others, or consider the contribution of their own behavior to the experience of others, leading to interpersonal conflicts.

### Summary of Integration Between Models

The main corollary that stems from our attempt to integrate theory and clinical practice in this area, is that discrete cognitive and behavioral impairments are likely to alter specific components of the RAM model. In this paper we have only underscored the relevance of considering how different forms of executive dysfunction can compromise a survivor's ability to reconnect with others. It is hoped that highlighting how specific impairments of executive function may impact specific processes of reaffiliation will lead to testable predictions in clinical practice and in further empirical research. However, it is sensible to consider that impairment of other non-executive skills (e.g., language, communication, memory, attention) could also alter the reaffiliation process. It is our belief that the systematic study of reaffiliation difficulties experienced by people with diverse profiles of neurocognitive disorders could offer valuable information to understand the neuropsychological bases of this process and its components. A summary of this adapted model including the contribution of executive skills at the different stages of the process can be found in Figure 2.

## Discussion

The main goal of this article was to advance our understanding of loneliness after brain injury by introducing the RAM model and theoretically exploring how different forms of executive impairment could alter the reconnection process generating persistent loneliness. This article proposes that diverse forms of cognitive impairment can alter different aspects of social interaction, such as social reconnection. Similar ideas have been proposed before, particularly by those interested in the relational impact of brain injury (Bowen et al., 2018). Yeates (2013), for example, has described how language, memory, attention or executive impairments can generate diverse forms of misattunement between couples. Yasmin and Riley (2021) have also shown that, in couples where one member had a brain injury, communication impairments predicted levels of relationship discontinuity. Working with people with dementia, Hydén (2017) has also reported how memory impairment can compromise couples' capacity to communicate and sustain a shared sense of identity (we-ness). This article attempts to contribute to this literature, as well as to the emerging literature on brain injury and loneliness, with a specific focus on executive impairment.

The emphasis on the relationship between neuropsychological impairments and difficulties in the reaffiliation process attempts to shed light on the many factors that can contribute to loneliness after brain injury. Even though we have focused on this variable here, other factors need to be considered as well. Negative bias during the *cognitive reaffiliation* phase may well be influenced by real experiences of social rejection and misunderstanding. It is well-known that brain injury, and its visible and non-visible sequelae, are poorly understood by people in the community (Code et al., 2016). This is often reported by survivors in experiences of feeling different from their previous self (Villa et al., 2021), misunderstood (Salas et al., 2018) or abnormal (Prigatano, 1999). There is also an emerging literature stressing how negative interactions can be internalized by survivors, leading survivors to conceal information about the injury to others (Hagger and Riley, 2017). There are also personal (biographical and temperamental) factors, not directly related to the injury that might contribute to a social negative bias, thus compromising reaffiliation (see tendency to experience negative affect -neuroticism, Rigon et al., 2019).

If executive impairment can compromise a survivor's capacity to reconnect to others when feeling lonely, other types of cognitive impairment should also have an impact in the reaffiliation process. Difficulties in social interaction, and social isolation, have been described as key long-term problems in people with aphasia, impacting quality of life and emotional adjustment (Code and Herrmann, 2003; Vickers, 2010). It has been described that people with aphasia have a less diverse network after the injury compared to older adults (Northcott and Hilari, 2011), and they tend to experience more negative interactions with others, and struggle more to maintain friendship, than people with brain injury but no aphasia (Northcott and Hilari, 2011). It is sensible to propose that comprehensive and expressive language abilities are fundamental to navigate through the reaffiliation process. Reduced conversation opportunities and increased communication difficulties (Blom Johansson et al., 2012) can frequently -and chronically- trigger feelings of loneliness (*perception of loneliness*). Communication ruptures are common after aphasia, but they can be satisfactorily dealt with if the person with aphasia uses appropriate strategies and is understood and supported by the communication partner (Dalemans et al., 2010). If communication ruptures are not repaired people with aphasia can start giving up or avoiding conversations. Thus, the non-reparation of these ruptures can contribute to negative biases toward social interaction, compromising cognitive and behavioral reaffiliation.

Deep or profound amnesia is another interesting type of impairment to consider. Profound amnesia is often caused by diencephalic lesions, after different forms of encephalitis, strokes of the posterior cerebral artery, hypoxia or tumors. The socio-emotional consequences of profound amnesia are less well-known, despite the long-term observations of historical cases. It is known, however, that people with deep amnesia are able to develop new interpersonal relationships, despite not being able to remember who the new person is or where they met him/her (Tranel and Damasio, 1993; Turnbull et al., 2006; Moore et al., 2017). In a review of the literature on emotional and social consequences of memory disorders, Tate, 2000) described a pattern characterized by the attenuation of emotional responses, absence of emotional distress and concern about present circumstances -or when present easily removed from the focus of the mind after a distraction- as well as a tendency for inertia-type behaviors. This pattern is interesting to consider under the light of the RAM model. People with profound amnesia are able to perceive and experience a wide range of human feelings, loneliness included. However, due to episodic memory impairment, they are not able to remember the real or mental events that trigger such feelings (Feinstein et al., 2010). As it has been widely reported in case studies, behavior loses its connection to the past and the future, becoming bound to the “present moment”.

## Clinical Implications

In principle, the RAM model could be generalized to any form of ABI. However, based on the type of ABI alone, it is difficult to infer functional outcome or cognitive sequelae with a high degree of confidence. Each ABI may result in a broad range of cognitive and emotional sequelae dependent on a multitude of pre and post morbid factors. The focus of the current paper related to executive functioning, which is common following many forms of ABI (e.g., traumatic injury, anoxia, cerebrovascular accidents and infection), which makes executive functions a good candidate for regulating loneliness across a range of brain injuries.

The primary clinical implication of this article highlights the need to consider how diverse types of cognitive impairments can impact social interaction, compromising reaffiliation and contributing to persistent loneliness. All clinicians involved in neurorehabilitation should routinely assess for markers of social isolation (e.g., number and frequency of social contacts, social support), paying particular attention to the survivor's subjective experience of loneliness. This could be completed using well-validated questionnaires such as the UCLA Loneliness scale. Loneliness has systematically emerged as a key variable predicting quality of life and depressive symptoms (Salas et al., 2021). Thus, case formulations need to consider and carefully explore social isolation and loneliness in particular: Are physical, cognitive or behavioral deficits contributing to the experience of loneliness? What components of the reaffiliation process are particularly compromised due to the specific cognitive and behavioral impairments of the survivor? Has he/she experienced traumatic interpersonal experiences since the injury, feeling misunderstood or attacked? Loneliness, and social isolation, should also be considered in the context of the individuals recovery journey. For instance, those in the acute stages of recovery may be initially overstimulated by increased social interactions. Therefore, social isolation may actually be beneficial in the early stages of recovery.

In addition, clinicians may complement the clinical interview using self-report measures to screen and quantify loneliness (Valtorta et al., 2016), such as the De Jong Gierveld Loneliness Scale (De Jong Gierveld and Van Tilburg, 1999) or the UCLA Loneliness scale (Russell et al., 1978). Clinicians often screen for depression and anxiety, but not loneliness despite the prevalence of loneliness being equal to, or indeed greater than, anxiety and depression (Byrne et al., 2022). Of course, loneliness, depression and anxiety highly correlate and it may be difficult to disentangle between these ovelaping constructs. These questions, together with information derived from third party informants and traditional neuropsychological assessment, can help the clinician to include social isolation and loneliness in dense case formulations. Only by including this information will it be possible to design interventions that can specifically target the factors that contribute to loneliness and persistent loneliness (e.g., cognitive and behavioral impairments, cognitive bias in the survivor, identity issues, environmental barriers, lack of information and training of relatives and friends, etc.).

Another clinical implication is the need to study and develop interventions that specifically address loneliness after brain injury. There is some evidence that enlarging social networks of survivors can reduce loneliness (Rowlands, 2002; Vickers, 2010; Northcott and Hilari, 2011). This is consistent with data showing an inverse relationship between network size and loneliness in people with brain injury (Salas et al., 2021). However, evidence has also demonstrated that objective aspects of social connectedness, such as relationship status, proximity of others and frequency of contact in stroke populations explain only a small amount of variance of subjective reported loneliness (Byrne et al., 2022). Therefore, it is reasonable to question whether interventions increasing social network size is enough, pointing at the need to combine it with interventions that tackle psychological (e.g., low self-esteem, avoidance) and neuropsychological (e.g., impulsivity, concreteness) difficulties, thus equipping the survivor with adequate tools to navigate social interaction and connect to others (Rigon et al., 2019). This article has attempted to contribute to this literature by proposing a model useful to understand the many factors that can exacerbate loneliness in individuals with ABI.

Depending on the neuropsychological profile of the survivor, clinicians could design specific interventions using the RAM model as a theoretical framework, thus targeting specific components of the process that may be particularly compromised. For example, those with specific energisation difficulties may struggle more at early stages of the reaffiliation process (perception of social isolation and activation of reaffiliation), than those with executive cognition difficulties, who may require support later along the process (cognitive reaffiliation). Classic neuropsychological rehabilitation strategies could be adapted to promote reaffiliation at different stages, since evidence-based interventions developed to manage different forms of executive impairment have been widely reported in the literature. People with energization problems could benefit from developing and sustaining routines that include social interaction as a central activity (Jackson et al., 2014). Prompting devices could also be a potentially useful compensatory strategy to remember social activities and initiate behavior (Jamieson and Evans, 2014). Using everyday collaborators as external motivation (Jamieson et al., 2020) to engage in social interactions in these groups could be also a beneficial intervention.

Brain injured survivors with executive cognition problems may benefit from learning strategies that enhance thinking skills when appraising a social situation or when solving a problem that emerges during an interpersonal encounter, thus avoiding confusion and perseveration. Goal Management Training (Levine et al., 2000), for example, is a tool that can help survivors make decisions and solve problems in non-structured situations. People with behavioral and emotional dysregulation could benefit from strategies that help them recognize early signs of rising emotions in social situations, in order to avoid catastrophic reactions (Ben-Yishay and Diller, 2011) and acting impulsively (Winegardner, 2017). Training in the use of calming cognitive and mood oriented strategies (Winegardner, 2017) can contribute to the necessary withholding of prepotent responses during reaffiliation, thus promoting the necessary “thinking space” to assess and consider all elements of a social situation before acting. Finally, several strategies have been developed to facilitate awareness for those with executive metacognitive impairments (Winegardner, 2017). The “zoom in/zoom out” strategy, for example, helps survivors to detach from immediate experience and consider the “larger picture” by imagining his/her mind as a camera lens. Feedback and behavioral experiments can be particularly useful to address metacognitive problems in social settings, since they can help identify negative beliefs (Bennett-Levy et al., 2004) or increase awareness of the survivor's own behavior and the impact that the survivor's behavior can have on others (Schmidt et al., 2011).

## Author Contributions

CB and CS conceived the presented idea linking existing models of executive function to a model of loneliness. CB, CS, RC, and RR further refined the presented ideas and contributed to the final manuscript. All authors contributed to the article and approved the submitted version.

## Funding

This research was performed as part of an all-Wales Economic and Social Research Council (ESRC) Doctoral Training Centre Ph.D. Studentship (awarded to RR and RC, Ph.D. student: CB).

## Conflict of Interest

The authors declare that the research was conducted in the absence of any commercial or financial relationships that could be construed as a potential conflict of interest.

## Publisher's Note

All claims expressed in this article are solely those of the authors and do not necessarily represent those of their affiliated organizations, or those of the publisher, the editors and the reviewers. Any product that may be evaluated in this article, or claim that may be made by its manufacturer, is not guaranteed or endorsed by the publisher.
